# Normal Speed and Accuracy of Saccade and Vergence Eye Movements in Dyslexic Reader Children

**DOI:** 10.1155/2009/325214

**Published:** 2010-01-19

**Authors:** Maria Pia Bucci, Marine Vernet, Christophe-Loïc Gerard, Zoï Kapoula

**Affiliations:** ^1^FRE 3292 CNRS, Université René Descartes Paris V, 71 avenue Edouard Vaillant, 92774 Boulogne Billancourt Cedex, France; ^2^FRE 3154 CNRS, Pôle Chirurgie ORL-OPH, Hôpital Robert Debré, 48 boulevard Sérurier, 75019 Paris, France; ^3^Service de Psychopathologie de l'Enfant et de l'Adolescent, Hôpital Robert Debré, 48 boulevard Sérurier, 75019 Paris, France

## Abstract

*Objective*. Latency of eye movements depends on cortical structures while speed of execution and accuracy depends mostly on subcortical brainstem structures. Prior studies reported in dyslexic reader children abnormalities of latencies of saccades (isolated and combined with vergence); such abnormalities were attributed to deficits of fixation control and of visual attention. In this study we examine speed and accuracy characteristics of horizontal eye movements in natural space (saccades, vergence and combined movements) in dyslexic reader children. *Methods*. Two paradigms are tested: gap paradigm (fixation offset 200 ms prior to target onset), producing shorter latencies, in both non-dyslexic reader and dyslexic reader children and simultaneous paradigm. Seventeen dyslexic reader children (mean age: 12 ± 0.08 years) and thirteen non-dyslexic reader children (mean age: 12 ± 1 years) were tested. Horizontal eye movements from both eyes were recorded simultaneously by a photoelectric device (Oculometer, Dr. Bouis). *Results*. For all movements tested (saccades, vergence, isolated or combined) and for both paradigms, the mean velocity and accuracy were similar in dyslexic readers and non-dyslexic readers; no significant difference was found. *Conclusion*. This negative but important result, suggests no dysfunction of brainstem ocular motor circuits in dyslexic readers. It contrasts results on latencies related to visual attention dysfunction at cortical level.

## 1. Introduction

During visual exploration of the natural world, humans make saccades, vergence, and combined saccade-vergence movements. The saccades are the fast eye movements used to change the direction of fixation rapidly; they are conjugate movements, that is, the eyes move equally and in the same direction. Vergence eye movements are necessary to change fixation from a far to a close target, or vice versa; these movements are disconjugate (in opposite direction in the two eyes). Combined movements are the movements most frequently made when looking at objects in the space needing a shift of gaze both in direction and in depth. To our knowledge, a fine exploration of the speed and accuracy characteristics of all these eye movements used during the everyday life has never been done in dyslexic readers. 

Indeed, the majority of the studies dealing with eye movements and dyslexia are limited to saccades made during reading or made towards isolated single target (tracking condition) [1]. Pavlidis [2] reported frequent regressive saccades and unstable fixation in dyslexia during reading and during sequential tracking of LEDs. Shorter saccade amplitude and longer fixation durations during reading have been reported [3, 4]. In contrast, Olson et al. [5] found no eye movement abnormalities in dyslexic readers when reading pseudowords and Hutzler et al. [6] also reported no eye movement differences between dyslexic and non-dyslexic reader subjects on a string-processing task or while reading pseudowords. Specifically, there were no differences in the number of fixations, or in the duration of the first fixation, or in the total reading time. Recently our group [7, 8] reported in dyslexic readers poor binocular coordination of saccades and of the postsaccadic fixation period not only during reading single words and during tracking condition but also during free exploration of artwork, suggesting that such problems occur independently from the reading process and might reflect immaturity of normal oculomotor learning mechanisms.

Another group of studies has examined the latency of saccades under various oculomotor conditions and at different viewing distances. Dossetor and Papaioannou [9] and Pirozzolo [10] observed longer saccade latency in dyslexic readers, while other authors [11, 12] did not find latency difference between dyslexic readers and non-dyslexic readers. Fischer and Weber [13] also examined saccade latency distribution in dyslexic readers and they reported longer mean latencies and large standard deviation in these children; moreover, other researchers [14] observed a high number of express saccades with the gap and with the overlap paradigms in dyslexic readers. 

All these findings suggested abnormal function of the fixation system in dyslexia, and abnormalities in saccade initiation and accuracy. Our group recently extended studies in dyslexic readers examining several types of eye movements in the natural far and near space [15]; we reported that the latency of saccades at far distance and of saccades combined with convergence was significantly longer in dyslexic readers; moreover, we found higher rates of express latency in dyslexic readers that occur not only for saccades but for other types of eye movements, particularly for divergence. Most likely, problems of both voluntary and reflexive controls of attention during eye movement preparation could explain the findings observed in dyslexic readers. 

Latency as well as fixation stability are known to be controlled by central and cortical structures; in contrast, speed and accuracy of eye movements are controlled by both cortical and premotor structures at the brainstem level where the motor command is prepared [16]. More precisely, the speed and the amplitude of the saccades depend on the properties of the saccade burst generator located for the horizontal direction at the paramedian pontine reticular formation (PPRF) in the brain stem [16]. The PPRF receives inputs from several cortical structures (frontal eye field, parietal cortex), the superior colliculus, and the cerebellum [16]. The neural organisation of the vergence brainstem generators looks like that of the saccade system. Indeed, neurons involved specifically in the control of vergence movements have been found in the mesencephalic reticular formation of monkeys [17–19]. Convergence and divergence burst neurons were found to discharge in relation to vergence velocity; moreover, [20, 21] provided evidence that the activity of neurons in the prearcuate area, close to the frontal eye field of monkeys was related to vergence dynamic.

Relatively little is known about the neuronal circuitry controlling combined movements. Some authors [22, 23] proposed a model in which combined eye movements could be produced by a coactivation of the two distinct oculomotor systems, that of the saccade and that of the vergence. The omnipause neurons (OPN) could gate the activity of a specific pool of neurons, the so-called saccade-related-vergence bursters responsible for generating both saccade and vergence commands. Chaturvedi and van Gisbergen [24] showed that the superior colliculus via the OPN controls the saccade and the vergence responses during combined movements. Indeed, when the target called for combined movement, electrical stimulation of the rostral region of the superior colliculus of monkeys inactivated both the saccadic and the vergence systems. 

Behavioural oculomotor studies carried out in children and in adults give us some information on the development state of such premotor structures. For instance, Cohen and Henn [25] showed that speed of saccades does not change significantly with age, suggesting that the PPRF is completed developed by the age of 4 years. Subsequent studies confirmed this results reporting that saccade speed in children of 4 years was similar to that observed in adults [26, 27] and that saccade amplitude was constant from the age of 12 years [26]. Yang and Kapoula [28] explored eye movements in natural space in children from the age of 4.5 years; they showed that saccades, vergence, and combined movements are similarly accurate as for adults (close to 90% of the amplitude required by the target) in children from the age of 4.5 years. Moreover, these authors did not found any significant difference in the speed of all types of eye movements between children and adults. These observations indicate early maturation of all distinct brainstem structures controlling eye movements (saccades and vergence) and their interaction. 

The goal of the present study was to explore further the speed and the accuracy characteristics of natural eye movements in 3D space in dyslexic children in order to find out whether some kind of dysfunction in the brainstem oculomotor circuits could be present in this population of children. Data from dyslexic readers were compared to those from an age-matched group of non-dyslexic reader children.

Furthermore, two paradigms (gap and simultaneous) were used to elicit eye movements in natural space. It is well known that the gap paradigm decreases the latency of eye movements [29]; and our prior study [15] showed in dyslexic reader children this effect for the latency of several types of eye movements (saccades, vergence, and combined movements). Here we want to examine whether speed and accuracy characteristics could also be affected by the gap paradigm and eventually to find out differences in the two groups of children (dyslexic and non-dyslexic readers). Our general objective was to contribute to enlarge knowledge on oculomotor behaviour in dyslexic reader children.

## 2. Materials and Methods

### 2.1. Subjects

Seventeen dyslexic reader children participated in the study. Dyslexic children were recruited from the pediatric hospital where they are referred for a complete evaluation of their dyslexia state with an extensive examination including neurological/psychological and phonological capabilities. For each child, the time of reading a text, its comprehension, and the capacity of reading word/pseudowords hve been evaluated by using the L2MA battery [30]. This is the standard test developed by the applied psychology centre of Paris, and is used everywhere in France. Inclusion criteria were scores on this test beyond 2 standard deviations and a normal mean intelligence quotient (IQ, evaluated with WISC III), that is, between 85 and 115. The mean age of the dyslexic children was 12 ± 0.08 years, the mean IQ was 100 ± 6, and the mean reading age was 9 ± 1 years. A carefully selected age-matched control group (mean age: 12 ± 1 years) of thirteen non-dyslexic children was selected. These children had to satisfy the following criteria: no known neurological or psychiatric abnormalities, no history of reading difficulty, and no visual stress or any difficulties with near vision. IQ and reading measurements were not available for these children, but they were selected by the director of the school on the basis of their school performances; their score on French (reading, understanding, and orthography), mathematic, and foreigner languages were all beyond the mean score of the class. Recruitment for controls, based on school performance alone has been used by others [31–33]. 

Both non-dyslexic and dyslexic reader children underwent an ophthalmologic examination accompanied by orthoptic evaluation of their visual function (median values showed in Table 1). All children had normal binocular vision (60 seconds of arc or better), that was evaluated with the TNO random dot test. Visual acuity was normal (≥20/25) for all children, dyslexic and non-dyslexic readers. The near point of convergence was abnormal (>8 cm) in 30% of dyslexic children while it was normal (≤7 cm) for all non-dyslexic children. Moreover, an orthoptic evaluation of vergence fusion capability using prisms and Maddox rod was done at far and at near distances. The divergence amplitude was limited in 27% and 76% of the cases for dyslexic readers, respectively, at far and at near distances; while for non-dyslexic children, limited divergence was observed only at near distance in 46% of the cases. Convergence amplitude was abnormal in 31% and 12% of dyslexic readers (at far and near at distance, respectively) and in the normal range for all but one non-dyslexic child at far distance. Phoria (i.e., latent deviation of one eye when the other eye is covered, using the cover-uncover test) was abnormal in 18% of dyslexic reader children at far as well as at near distances; while for non-dyslexic children, phoria was abnormal in 15% and in 7% at far and at near distance, respectively. 

In sum, orthoptic evaluation showed a tendency of poor divergence amplitude particularly at near distance in dyslexic reader children in line with another study on a larger population of dyslexic and non-dyslexic reader children [34].

The investigation adhered to the principles of the Declaration of Helsinki and was approved by our Institutional Human Experimentation Committee. Informed consent was obtained from the children's parents after the procedure for the experiment was explained. 

Note that the majority of children, dyslexic and non-dyslexic readers, participated already to our previous study [15] in which only eye movement latencies have been analyzed.

### 2.2. Oculomotor Paradigm

**Figure 1:**
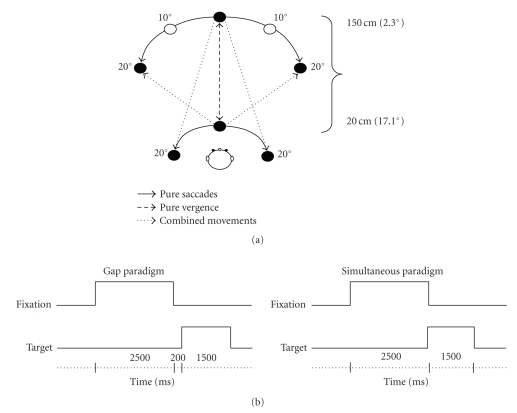
Spatial arrangement (a): LEDs were placed on an horizontal table at eye level. Three types of eye movements were elicited depending on the combination of the fixation and target LEDs: pure saccades far or close, pure vergence along the median axis, and combined movements; Temporal arrangement (b); schematic diagram of the temporal arrangement used in the two different paradigms (gap and simultaneous).

The spatial and the temporal arrangement are the same to that used in our previous study [15]. Briefly, eight LEDs were embedded in two isovergence circles at different distance (20 and 150 cm) on an horizontal table (see Figure 1(a)). Five LEDs were placed 150 cm from the subjects' eyes, one at the center, two at ±10°, and two at ±20°. The required mean angle of vergence for fixating these diodes was 2.3°. The other three LEDs were placed at a distance of 20 cm, one at the center and two at ±20°; the mean angle of vergence was 17.1°. Three types of movements were elicited: pure saccades, pure vergence, and combined movements. The fixation point was either the central LED at the distance of 20 cm or the central LED at the distance of 150 cm. Pure saccades to the left or to the right were elicited either at a close distance of 20 cm or at a far distance of 150 cm. Pure vergence was either convergence or divergence between the two LEDs placed on the median plane at 20 cm and 150 cm. Combined eye movements involved changes both in direction and in depth. The required saccade amplitude was always 20° for both pure saccades and combined movements. The required vergence movement was always 14.8° (17.1 − 2.3°) for both pure vergence along the median plane and combined eye movements.

Two temporal paradigms were used (gap and simultaneous, see Figure 1(b)). The gap paradigm was used to elicit short-latency eye movements. For each trial, the central LED (at a distance of either 20 cm or 150 cm) was switched on for a period of 2.5-second. Then it was switched off, and a target LED appeared 200 ms later (gap period). The target LED stayed on for 1.5 seconds. A delay of 0.5 seconds was introduced before the next trial. The simultaneous paradigm was used to elicit more voluntary eye movements. After a 2.5 seconds fixation period, the central LED was switched off, and simultaneously the target LED was switched on for 1.5 seconds. A delay of 0.5 seconds was introduced before the next trial. The instruction given to the children was to look at the target LED as accurately and as rapidly as possible.

### 2.3. Procedure

Children were in a dark room and faced the horizontal table; viewing was binocular. Child performed 4 blocks of 36 trials each, two with the gap and two with the simultaneous paradigm; each block was separated by a few minutes of rest. In each block the three types of trials were interleaved randomly. Calibration was done before and after each block to allow accurate evaluation of the amplitude of the saccades. Calibration factors for each eye were extracted from the eye positions during the calibration procedure; a polynomial function with five parameters was used to fit the calibration data.

### 2.4. Calibration Task

A standard saccade paradigm was used to elicit visually guided saccades to target-LEDs presented at 0°, ±10°, and ±20° at the far isovergence surface. The child fixated the central LED for 2 seconds; the central LED disappeared and another LED appeared for 2 seconds at an eccentric position to the left or to the right at 10° or 20°. Child was instructed to fixate the LED as accurately as possible; the LED presentation was sufficiently long to allow accurate fixation. These saccades were used to extract the calibration factor for each eye.

### 2.5. Eye Movements Recording

Data collection was controlled by REX, a software developed for real-time experiments and run on a PC. Horizontal eye movements from both eyes were recorded simultaneously with a photoelectric device (Oculometer, BOUIS, Karlsruhe, Germany). This system has a resolution of 2 minutes of arc and a linear range of 20°. There is no obstruction of the visual field with this recording system [35]. Eye-position signals were lowpass filtered with a cutoff frequency of 200 Hz and digitized with a 12-bit analogue-to-digital converter; each channel was sampled at 500 Hz.

### 2.6. Data Analysis

A polynomial function with five parameters was used to calibrate individual eye position signal. From these two signals, we derived the conjugate saccadic signal [(left eye + right eye)/2] and the vergence disconjugate signal (left eye − right eye). Off-line computer algorithms based on standard velocity and acceleration criteria were used to determine the saccade and vergence onset and offset. The onset of the conjugate saccadic component (for pure saccades and for the saccade component of combined movements) was defined as the time when the eye velocity reached 5% of the saccadic peak velocity; the offset of this signal was defined as the time when the eye velocity dropped below 10°/s. The onset and the offset of the vergence signals (for pure vergence movements and for the vergence component of combined movements) were defined as the time point when the eye velocity exceeded or dropped 5°/s. These criteria are standard and similar to those used by other authors [36]. 

For all types of eye movements and for both saccade and vergence components, we measured the gain, which is the ratio of the amplitude of the total movement over the target excursion amplitude and the mean velocity (amplitude of the movement over duration). Statistical analysis was performed by the two-way ANOVAs as with between subject factor of the two groups of children (dyslexic readers and non-dyslexic readers) and as within subject factor of the individual means of the mean velocity and of the gain for each type of eye movements for the two paradigms used (gap and simultaneous). 

## 3. Results

### 3.1. Mean Velocity

**Figure 2:**
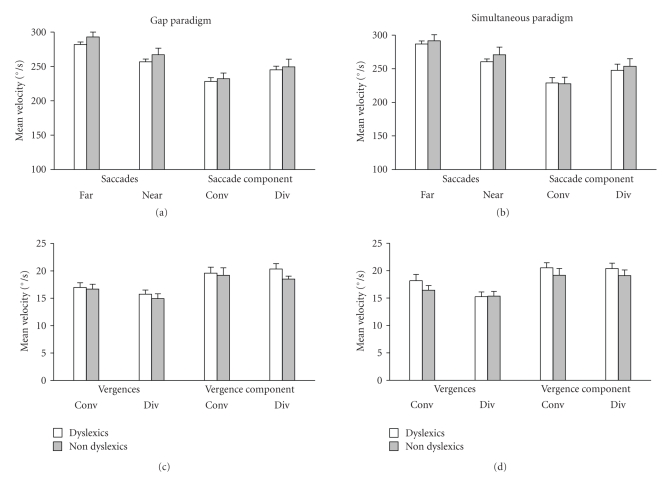
Mean velocity (amplitude of the movement/duration) of different types of eye movements in the gap (a) and (c) and in the simultaneous paradigm (b) and (d) for dyslexic (white bars) and non-dyslexic reader (gray bars) children. Vertical lines indicate standard error.

Figure 2 shows the mean velocity of all eye movements tested in the gap and in the simultaneous paradigm for the two groups of children (dyslexic and non-dyslexic); in Figures 2(a) and 2(b), movements with high mean velocity, saccades in pure and combined form are shown, and in Figures 2(c) and 2(d), movements with slow mean velocity (vergence pure and combined with saccades) are shown. Table 2 shows the minimum and the maximum mean velocity values for dyslexic and non-dyslexic reader children for each type of eye movements examined in the two paradigms. The mean velocity value for each type of eye movements examined in dyslexic readers is similar to that found in non-dyslexic readers. For each type of eye movements, the ANOVA does not show any significant difference between the two groups of children (F_(1,28)_ = 1.43, *P* = .24); there is no significant effect of the paradigms and there is no significant interaction between the groups of children and the paradigms. In sum, speed of eye movements in dyslexic reader is normal.

### 3.2. Accuracy

**Figure 3:**
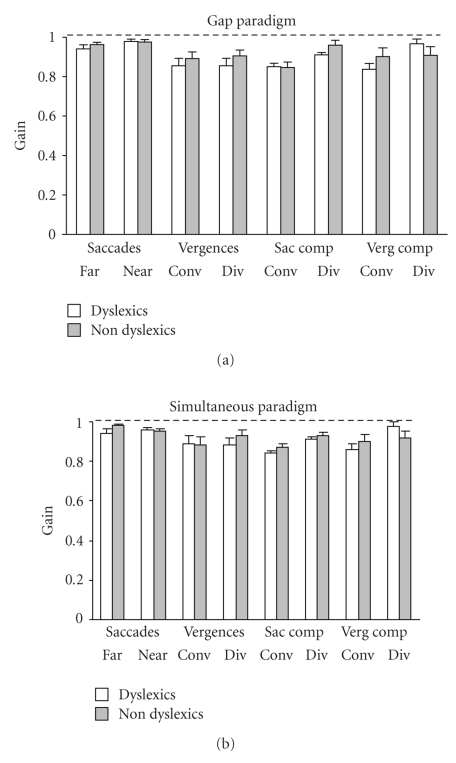
Mean gain (amplitude of the movement/target excursion) of different types of eye movements in the gap and in the simultaneous paradigm for dyslexic (white bars) and non-dyslexic reader (gray bars) children. Vertical lines indicate standard error.

Figure 3 shows the mean gain (amplitude of the movement/target excursion) of all types of eye movements tested (saccades at far and at near distance, convergence, divergence, and both components of combined saccade-vergence movements) in the gap and in the simultaneous paradigms for dyslexic and non-dyslexic reader children. Table 3 shows the minimum and the maximum gain values for each group of children for each type of eye movements examined in the two paradigms tested. For each type of eye movements examined, the mean gain in dyslexic readers is similar to that found in non-dyslexic readers. For each type of eye movements, the ANOVA does not show any significant difference between the two groups of children (F_(1,28)_ = 1.74, *P* = .19); there is no significant effect of the paradigms and there is no significant interaction between the groups of children and the paradigms. In conclusion, eye movements in dyslexic readers are as precise as in non-dyslexic readers.

## 4. Discussion

This study shows that the mean velocity and the accuracy of eye movements in natural space (saccades, vergence, and combined eye movements) in dyslexic readers are as good as in non-dyslexic children of similar age. These findings are new and contribute to know further the oculomotor capabilities in a population of dyslexic readers. Indeed, while studying latency of eye movements provides information about cortical function, speed-accuracy parameters are important measures to understand the functioning of the premotor and central circuits involved in the triggering of eye movements [16]. Our previous study conducted on dyslexic reader children [15] showed abnormally longer latency for some types of eye movements suggesting that such population of children has some difficulties on both voluntary and reflexive controls of attention. In contrast, the present study shows similar speed-accuracy characteristics in dyslexic and non-dyslexic reader children. Based on all these findings, we can advance the hypothesis that dyslexic reader children have problems in the central and cortical processes involved in the preparation of eye movements while the premotor and central areas responsible of the triggering of such eye movements are working as well as in non-dyslexic readers. This idea is also in line with our findings on binocular control of saccades and fixation in dyslexic readers [7, 8]. Indeed, in these studies we reported that dyslexic children showed poor quality of binocular coordination of saccades and fixation, independently of the task used, suggesting an intrinsic ocular motor deficit. Such a deficiency could be related to immaturity of the normal ocular motor learning mechanisms via which ocular motor coordination and stable fixation are achieved. The cerebellum and cortical areas of the magnocellular stream such as the parietal cortex could be the sites of ocular motor learning [16]. Consequently, we suggest that dyslexic reader children may have immaturity and/or deficiency in this network. In the future, studies examining further adaptive cortical learning mechanisms alone and combined with visual attention training activities in dyslexic and non-dyslexic children are needed to improve the knowledge on dyslexia.

Finally, this study also shows that temporal manipulation of the stimulus presentation (by using gap and simultaneous paradigms) does not influence the speed and the accuracy characteristics of eye movements for the two populations of children here tested (dyslexic readers as well as non-dyslexic readers). The majority of studies dealing with gap effect reported only data on latency of eye movements, showing the decrease of latency by the gap paradigm [37]; the work of Pratt [38] examined both the latency and the kinematic features as saccadic amplitude, duration, average velocity, peak velocity, and peak acceleration in young adults. Results showed greater peak velocities for saccades elicited by the gap paradigm with respect to the overlap paradigm. Such result is in contrast with the data presented here; however, as also suggested by the author, these differences could be due to the specific experimental set up employed in the study (e.g., presence of a warning tone on every trial, limited number of subjects tested, overlap and gap paradigm compared) that was different to those used here. 

In conclusion, our data found out from a larger population of children (both dyslexic and non-dyslexic reader) suggest that speed and accuracy characteristics of eye movements are predominantly determined by brainstem structures and they are not influenced by temporal manipulation of the stimulus presentation; while the gap paradigm acts on physiological cortical mechanisms subtending attention and motor preparation leading to change in eye movement's latency.

## Figures and Tables

**Table 1:** Clinical characteristic of children.

	Stereo acuity (TNO)	NPC (cm)	Hetero phoria (pD)	Divergence (pD)	Convergence (pD)
Dyslexic readers (17)	60′′	5	far ortho	far 4	far 16
			near ortho	near 12	near 30
Non-Dyslexic readers (13)	60′′	5	far ortho	far 6	far 14
			near 3×	near 17	near 28

**Table:** (a)

	Dyslexic reader		Non-Dyslexic reader	
	min	max	min	max
Sac far	255	321	232	358
Sac near	200	299	208	312
Conv	11	22	11	21
Div	11	20	11	20
Sac comb conv	191	273	189	281
Sac comb div	207	280	163	330
Conv comb	13	26	13	28
Div comb	14	22	14	20

**Table:** (b)

	Dyslexic reader		Non-Dyslexic reader	
	min	max	min	max
Sac far	259	342	248	387
Sac near	211	283	204	328
Conv	10	24	11	21
Div	10	20	11	21
Sac comb conv	182	298	187	311
Sac comb div	170	290	188	342
Conv comb	14	26	13	25
Div comb	13	24	12	24

**Table:** (a)

	Dyslexic reader		Non-Dyslexic reader	
	min	max	min	max
Sac far	0.78	1.05	0.90	1.00
Sac near	0.90	1.05	0.90	1.00
Conv	0.67	1.03	0.70	1.02
Div	0.63	1.05	0.70	1.10
Sac comb conv	0.79	1.05	0.74	0.96
Sac comb div	0.82	1.00	0.83	1.10
Conv comb	0.67	1.00	0.70	1.00
Div comb	0.83	1.10	0.70	1.00

**Table:** (b)

	Dyslexic reader		Non-Dyslexic reader	
	min	max	min	max
Sac far	0.82	1.05	0.93	1.03
Sac near	0.89	1.05	0.90	1.00
Conv	0.65	1.13	0.66	1.10
Div	0.63	1.04	0.70	1.05
Sac comb conv	0.74	1.00	0.74	0.96
Sac comb div	0.84	1.00	0.80	1.00
Conv comb	0.67	1.00	0.72	1.00
Div comb	0.73	1.10	0.76	1.00
